# L-Rhamnose induction of *Aspergillus nidulans *α-L-rhamnosidase genes is glucose repressed via a CreA-independent mechanism acting at the level of inducer uptake

**DOI:** 10.1186/1475-2859-11-26

**Published:** 2012-02-21

**Authors:** Juan A Tamayo-Ramos, Michel Flipphi, Ester Pardo, Paloma Manzanares, Margarita Orejas

**Affiliations:** 1 Instituto de Agroquímica y Tecnología de Alimentos, Consejo Superior de Investigaciones Científicas, Agustín Escardino 7, 46980 Paterna, Valencia, Spain; 2 Present address: Fungal Systems Biology, Laboratory of Systems and Synthetic Biology, Wageningen University, Dreijenplein 10, 6703 HB Wageningen, The Netherlands

**Keywords:** *Aspergillus nidulans*, Carbon catabolite repression, CreA-independent, Inducer exclusion, α-L-rhamnosidase, Transcriptional regulation

## Abstract

**Background:**

Little is known about the structure and regulation of fungal α-L-rhamnosidase genes despite increasing interest in the biotechnological potential of the enzymes that they encode. Whilst the paradigmatic filamentous fungus *Aspergillus nidulans *growing on L-rhamnose produces an α-L-rhamnosidase suitable for oenological applications, at least eight genes encoding putative α-L-rhamnosidases have been found in its genome. In the current work we have identified the gene (*rhaE*) encoding the former activity, and characterization of its expression has revealed a novel regulatory mechanism. A shared pattern of expression has also been observed for a second α-L-rhamnosidase gene, (AN10277/*rhaA*).

**Results:**

Amino acid sequence data for the oenological α-L-rhamnosidase were determined using MALDI-TOF mass spectrometry and correspond to the amino acid sequence deduced from AN7151 (*rhaE*). The cDNA of *rhaE *was expressed in *Saccharomyces cerevisiae *and yielded *p*NP-rhamnohydrolase activity. Phylogenetic analysis has revealed this eukaryotic α-L-rhamnosidase to be the first such enzyme found to be more closely related to bacterial rhamnosidases than other α-L-rhamnosidases of fungal origin. Northern analyses of diverse *A. nidulans *strains cultivated under different growth conditions indicate that *rhaA *and *rhaE *are induced by L-rhamnose and repressed by D-glucose as well as other carbon sources, some of which are considered to be non-repressive growth substrates. Interestingly, the transcriptional repression is independent of the wide domain carbon catabolite repressor CreA. Gene induction and glucose repression of these *rha *genes correlate with the uptake, or lack of it, of the inducing carbon source L-rhamnose, suggesting a prominent role for inducer exclusion in repression.

**Conclusions:**

The *A. nidulans rhaE *gene encodes an α-L-rhamnosidase phylogenetically distant to those described in filamentous fungi, and its expression is regulated by a novel CreA-independent mechanism. The identification of *rhaE *and the characterization of its regulation will facilitate the design of strategies to overproduce the encoded enzyme - or homologs from other fungi - for industrial applications. Moreover, *A. nidulans *α-L-rhamnosidase encoding genes could serve as prototypes for fungal genes coding for plant cell wall degrading enzymes regulated by a novel mechanism of CCR.

## Background

The degradation of plant cell wall polysaccharides (i.e. cellulose, hemicellulose and pectins), and the subsequent utilization of their components as carbon sources is a key and highly regulated event when filamentous fungi grow on these substrates or infect plants. A number of those plant-derived substrates contain the neutral sugar L-rhamnose (6-deoxy-L-mannose) which is a component of the plant cell wall pectic polysaccharides rhamnogalacturonan I and rhamnogalacturonan II [1] and diverse secondary metabolites, including anthocyanins, flavonoids and triterpenoids (see reviews [2,3], and references therein). In addition, L-rhamnose (hereafter rhamnose) is present in animal tissues and in viruses, and is also a component of bacterial polysaccharides where it plays an important role in pathogenicity. The utilization, transformation or detoxification of these rhamnose-containing compounds involves different α-L-rhamnosidases (EC 3.2.1.40), which catalyze the hydrolysis of terminal non-reducing rhamnose residues in polysaccharides and α-L-rhamnosides including the artificial substrate *p-*nitrophenyl-α-L-rhamnopyranoside (*p*NPR) that is widely used to evaluate their activity. α-L-Rhamnosidases have been found in some plant and animal tissues as well as in a plethora of microorganisms including filamentous fungi (see reviews [2,3] and references therein). Based on primary sequence similarities these enzymes are classified within the Carbohydrate-Active Enzymes (CAZy) database [4] (http://www.cazy.org/) into three glycosyl hydrolase (GH) families. Whereas bacterial α-L-rhamnosidases are classified into either GH78 or GH106, fungal α-L-rhamnosidases (with the possible exception of the *Aspergillus niger *rhamnogalacturonan α-L-rhamnopyranohydrolase B [RgxB; EC 3.2.1.40] which is assigned to family GH28) belong to GH78.

α-L-Rhamnosidases are of considerable interest given their suitability in various applications within the food (e.g. citrus juice debittering, liberation of aromas and bioactive compounds), pharmaceutical (e.g. biotransformation of antibiotics and steroids), agro-/forestry (e.g. detoxification of rhamnose-conjugated plant secondary metabolites) and chemical industries (e.g. to produce rhamnose) ([2] and references therein). The biotechnological potentiality of these enzymes has led to the characterization of fourteen microbial α-L-rhamnosidase (GH78 family) encoding genes within the last decade (Additional file 1: Table S1). Of these only four were isolated from filamentous fungi: the genes encoding RhaA and RhaB of *Aspergillus aculeatus *[5], that encoding AkRha78 of *Aspergillus kawachii *[6] and the gene encoding the *Aspergillus nidulans *AN10277 protein [7], hereafter named *rhaA*.

In the presence of rhamnose as the sole carbon source, *A. nidulans *secretes at least one α-L-rhamnosidase of molecular mass ~102 kDa. Its biochemical properties make it suitable for oenological applications [8,9] as well as for improvement of the bioavailability of plant rhamnose-containing bioactive compounds. While this enzyme is active against naringin and hesperidin (major rhamnosylated flavonoids in citrus juices in which the rhamnose molecule is bound to glucose by α-(1,2) or α-(1,6) linkages respectively) and also the synthetic substrate *p*NPR (in which the aglycon is directly linked to the C1 position of rhamnose) [8,9], recombinant AN10277-His_6_/RhaA-His_6 _was not [7], strongly suggesting that these are two distinct enzymes. At least eight loci putatively encoding α-L-rhamnosidases have been annotated in the *A. nidulans *genome the products of which range from 62 to 169 kDa [10,11]. The locus encoding the α-L-rhamnosidase of oenological interest is however unknown.

The regulation of α-L-rhamnosidase genes is less well documented than that of many other genes encoding glycosyl hydrolases. It has been reported [12] that the *Lactobacillus plantarum ram1 *and *ram2 *genes are induced by rhamnose and repressed by D-glucose (hereafter glucose). However, the regulatory circuits controlling fungal α-L-rhamnosidase gene expression are unknown. In filamentous fungi it has been shown that the synthesis of a number of plant cell wall-degrading enzymes is regulated at the level of transcription i.e. induced when the plant polysaccharide or its degradation products are present and repressed by glucose (see reviews [13,14]). Glucose repression is a well-studied regulatory circuit in *A. nidulans *(reviewed in [15,16]), and the role of the wide-domain transcriptional repressor CreA in the expression of a number of genes encoding plant cell wall degrading enzymes has been widely characterized. Our previous studies [8] have shown that α-L-rhamnosidase activity in *A. nidulans *is induced by rhamnose and repressed by glucose. However, unlike other glycosyl hydrolase systems, relief of repression by glucose either by cultivation in the presence of the inducer and a so-called non-repressing carbon source (e.g. D-lactose or L-arabinose) or by the use of the *creA^d^30 *derepressed strain [17] was not sufficient for α-L-rhamnosidase production to be totally derepressed. It was therefore suggested that carbon catabolite repression (CCR) of *A. nidulans *α-L-rhamnosidase genes could involve a CreA-independent mechanism [8].

Here we show that rhamnose and glucose regulate α-L-rhamnosidase production at the transcriptional level in the model filamentous fungus *A. nidulans*. We have identified the *A. nidulans *gene encoding the previously characterized α-L-rhamnosidase [8,9] and confirmed that glucose repression of its expression, as well as that of the gene coding for the α-L-rhamnosidase AN10277/RhaA (*rhaA*), occurs by means of a CreA-independent mechanism. The data suggest that glucose prevents the entry of the inducing carbon source rhamnose into the cell and that this phenomenon does not involve CreA function. We thus hypothesize the existence of an inducer exclusion mechanism by which fungal α-L-rhamnosidase genes are controlled. In this regard, the lack of protection against the toxicity of the non-metabolizable glucose analogue 2-deoxy-D-glucose by a range of rhamnose concentrations suggesting that inducer exclusion is not due to competition for uptake of the inducer. Moreover, α-L-rhamnosidase gene expression was found to be repressed by sugars previously considered non-repressive as well as by some non-sugar carbon sources (e.g. ethanol). In contrast to glucose, ethanol did not prevent the uptake of rhamnose. Hence, these findings provide evidence for the existence of novel molecular mechanisms for exerting CCR on genes encoding plant cell wall degrading enzymes in filamentous fungi which could be of utility in modulating α-L-rhamnosidase production.

## Results and discussion

### The *A. nidulans *locus AN7151 encodes an α-L-rhamnosidase of potential oenological relevance

**Figure 1:**
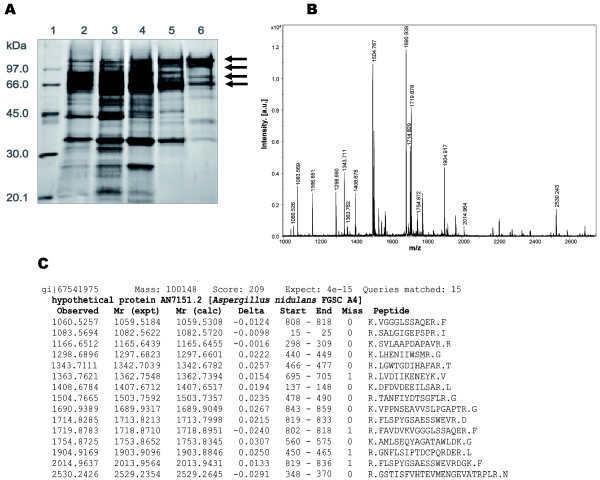
**Identification of the *A. nidulans *α-L-rhamnosidase gene by MALDI-TOF mass spectrometry**. (**A**) Silver stained SDS-PAGE of the α-L-rhamnosidase enriched fractions selected after each purification step. Lane 1, molecular mass markers; lane 2, crude extract (12 μg); lane 3 DEAE Sephadex A-50 (fraction 2, 43 μg); lanes 4 and 5, Q-Sepharose Fast Flow (fractions 20-22 and fractions 20-23 from two consecutive chromatographies, 17.5 and 3.5 μg, respectively); lane 6, Resource S (fractions 14-18; 2.5 μg). Molecular masses (kDa) are shown in the left margin. (**B**) Representative MALDI-TOF spectrum of one of the indicated protein bands (arrowed) from panel A. (**C**) Identification details for the AN7151 encoded protein from MASCOT searches.

With a view to identifying the *A. nidulans *gene encoding the α-L-rhamnosidase previously characterized [8,9], we determined the sequence of this protein. α-L-Rhamnosidase was purified from a culture filtrate of the *A. nidulans *wild-type strain (*biA1*) grown on 1% w/v rhamnose as the sole carbon source. The purification scheme [9] mainly consisted of two anion and one cation exchange chromatographic steps. Protein samples from each stage of the purification were resolved using SDS-PAGE (Figure 1A). Four major bands of ~66 to > 90 kDa were finally observed and processed for mass fingerprint analysis using MALDI-TOF mass spectrometry. Figure 1B shows the MALDI-TOF mass spectrum of a tryptic digest of one selected band. Representative peptide mass fingerprints for fifteen of the peptides are shown (Figure 1C). Comparison of the mass spectrometry data with the ORFs predicted in the *A. nidulans *genome (http://www.broad.mit.edu) identified locus AN7151, designated *rhaE*, as the encoding gene. The majority of the peptide sequences obtained from the four SDS-PAGE bands analyzed were identical, suggesting that these bands arose from proteolysis and/or deglycosylation of the purified enzyme.

### Gene structure of *A. nidulans rhaE*

Using RT-PCR a full-length cDNA corresponding to the ORF of the *A. nidulans rhaE *gene was generated and sequenced. Comparison of the cDNA sequence with the genomic sequence (Additional file 2: Figure S1) revealed the presence of two introns of 59 and 51 nucleotides which followed the canonical GT-AG splicing rule (E357/V358 and N504/Y505 within the bacterial alpha-L-rhamnosidase domain - Pfam PF05592 - of the *A. nidulans *predicted protein). The second intron is in-frame which explains its absence in automatic annotation of the gene. Glycosyl hydrolase genes are frequently found to be subjected to CCR and have CreA consensus motifs upstream of the ORF. Sequence analysis of the 1 kb sequence upstream of the proposed translational start site revealed the presence of 8 CreA (5'-SYGGRG-3') consensus target sites [18-20], 6 of them being located within the first 360 bp, suggesting that the transcription of *rhaE *could be directly repressed by CreA. However, it has previously been shown that the production of this α-L-rhamnosidase is repressed predominantly via a CreA-independent mechanism [8] which would indicate that the putative CreA targets in *rhaE *are not functional. Such a circumstance would not be without precedent for genes encoding plant cell wall degrading enzymes [21].

The polypeptide encoded by *rhaE *is 861 amino acids (aa) long and has a calculated molecular mass of 95205 Da. This value is in excellent agreement with the estimated molecular mass of the deglycosylated form of the purified α-L-rhamnosidase. The glycosylated form had a molecular mass of 102 kDa of which 7% was contributed by carbohydrates [9]. Intriguingly, while the *A. nidulans *α-L-rhamnosidase activity was detected and purified from the culture medium, the predicted RhaE protein appears to lack an N-terminal signal peptide (SignalP probability 0.081), suggesting that secretion of this enzyme may occur through a non-classical pathway [22]. In this regard, subcellular localization prediction using the SecretomeP program supports (NN/SecP score 0.608) this possibility.

The crystal structure of *Bacillus *sp. GL1 α-L-rhamnosidase RhaB has been resolved, and mutational studies have shown that four acidic residues (D567, E572, D579 and E841) are crucial for catalysis and/or substrate binding [23]. All four residues (D458, E464, D471 and E737) are conserved in the *A. nidulans *RhaE protein (Additional file 3: Figure S2) and are hence candidates for roles of functional importance.

### Phylogenetic relationships between the *A. nidulans *RhaE and class A bacterial α-L-rhamnosidases

**Figure 2:**
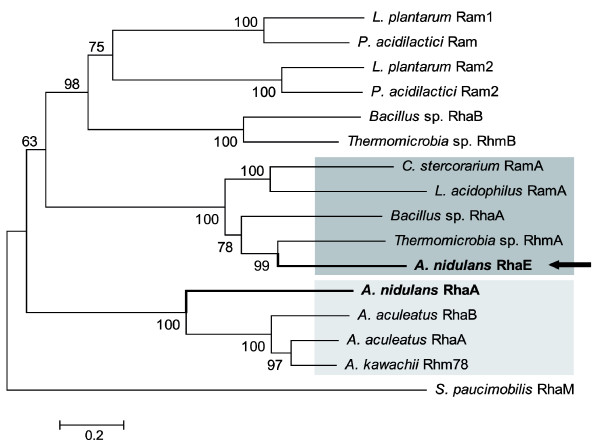
**Neighbour-Joining tree showing the phylogenetic relationships of the *A. nidulans *rhamnosidases RhaA and RhaE**. MEGA3 software [24] was used to carry out the phylogenetic analysis. Amino acid sequences were aligned and the dendogram was determined using ClustalW and the Neighbour-Joining algorithm. Bootstrap values adjacent to each internal node represent percentages of 1000 replicates. The scale bar represents aa replacements per site. Sequences from the following UniProtKB database accessions were included: *A. aculeatus *Q9C1M9 (RhaA), *A. aculeatus *Q9HFW5 (RhaB), *A. kawachii *A9ZT55 (Rhm78), *A. nidulans *C8VMJ6 (AN10277/RhaA), *Bacillus *sp. GL1 Q93RE8 (RhaA), *Bacillus *sp. GL1 Q93RE7 (RhaB), *C. stercorarium *Q9S3L0 (RamA), *L. acidophilus *Q5FJ31 (RamA), *L. plantarum *Q88SF8 (Ram1), *L. plantarum *Q88SF6 (Ram2), *Pediococcus acidilactici *EONEV1 (Ram), *P. acidilactici *EONEK0 (Ram2), *Thermomicrobia *sp. PRI-1686 Q93RE8 (RhmA) and *Thermomicrobia *sp. PRI-1686 Q93RE7 (RhmB). *Sphingomonas paucimobilis *RhaM (Q76LC4), a GH106 rhamnosidase, was used to root the tree.

A phylogenetic tree based on aa sequence homologies was constructed for *A. nidulans *RhaE and those GH78 proteins encoded by experimentally verified genes (Figure 2). Alignment (ClustalW) was carried out with the program MEGA 3 [24] and the Neighbour-Joining algorithm with bootstrap values from 1000 trials was utilized. Interestingly, *A. nidulans *RhaE resides in a well-defined clade (boostrap value 100) containing the four class A bacterial α-L-rhamnosidases (i.e. *Clostridium stercorarium *RamA, *Lactobacillus acidophilus *RamA, *Bacillus *sp. GL1 RhaA and *Thermomicrobia *sp. PRI-1686 RhmA) whereas all other fungal α-L-rhamnosidases, including *A. nidulans *RhaA, segregated together in a highly divergent branch. This fungal cluster contains shorter enzymes (597-661 aa residues) whereas enzymes of the RhaE cluster are at least 200 residues longer (861-932 aa).

### Conservation of RhaE among fungi

As RhaE is the first eukaryotic enzyme with structural similarity to bacterial α-L-rhamnosidases, we screened the fungal genome databases to see whether RhaE homologs could be found in other fungal species. BLASTP [25] searches using the RhaE sequence as a query confirmed its conservation in both the Ascomycota and Basidiomycota (Table 1). The most similar ortholog (69% identity over 869) was found in *Aspergillus terreus *and could correspond to the oenological α-L-rhamnosidase previously purified [26]. Orthologs with at least 50% identity were identified in *Aspergillus flavus, Aspergillus oryzae, Magnaporthe grisea, Nectria haematococca, Fusarium graminearum, Fusarium verticilloides *and *Fusarium oxysporum*. Less similar proteins (40-49% identity) were found in *Verticillium albo-atrum, Penicillium chrysogenum, Aspergillus niger, Neosartorya fischeri, Tuber melanosporum, Penicillium marneffei, Aspergillus fumigatus, Stagonospora nodorum*, the hemiascomycete yeast *Clavispora lusitaniae *and the Basidiomycetes *Cryptococcus neoformans *and *Cryptococcus gatii*. The *A. nidulans *genome specified two additional loci (AN12368 and AN11954) that could encode paralogs of RhaE (48% and 45% identity over 869 and 863 aa respectively). Additional studies are required to determine the catalytic activities of their products.

**Table 1:** Selected fungal homologs of the *A. nidulans *AN7151/RhaE rhamnosidase


**Organism**	**Accession no./Reference ** (NCBI/Broad Institute*)	***E *value**	**% Identity/% Similarity/** **Aligned amino acids**

**ASCOMYCOTA**			
**Eurotiomycetes**			
*Aspergillus terreus*	XP 001212100	0.0	69/81/869
*Aspergillus oryzae*	XP 001827536	0.0	57/70/867
*Aspergillus flavus*	XP 002384747	0.0	56/69/869
*Penicillium chrysogenum*	XP 002560426	0.0	46/62/889
*Aspergillus nidulans*	XP 658315	0.0	48/63/869
*Aspergillus niger*	XP 001402266	0.0	45/62/893
*Aspergillus nidulans*	XP 662910	0.0	45/62/863
*Neosartorya fischeri*	XP 001258340	0.0	46/61/873
*Penicillium marneffei*	XP 002152078	0.0	47/64/807
*Aspergillus fumigatus*	XP 751335	0.0	43/59/886
**Dothideomycetes**			
*Stagonospora nodorum*	SNOG_14400*	0.0	40/58/885
**Sordariomycetes**			
*Magnaporthe grisea*	XP 365490	0.0	54/70/876
*Nectria haematococca*	XP 003041639	0.0	55/71/863
*Fusarium verticillioides*	FVEG_12337*	0.0	54/70/866
*Fusarium oxysporum*	FOXG_13192*	0.0	53/70/866
*Fusarium graminearum*	FGSG_07974*	0.0	53/69/869
*Verticilium albo-atrum*	XP 003009324	0.0	49/65/867
**Pezizomycetes**			
*Tuber melanosporum*	XP 002839085	0.0	45/63/853
**Saccharomycetes**			
*Clavispora lusitaniae*	XP 002617481	4e^-165^	40/57/863
**BASIDIOMYCOTA**			
**Tremellomycetes**			
*Cryptoccocus neoformans*	XP 571766	0.0	42/58/876
*Cryptoccocus gattii*	CNBG_5170	0.0	43/61/769

### Functional expression of *A. nidulans *RhaE α-L-rhamnosidase in *S. cerevisiae*

**Figure 3:**
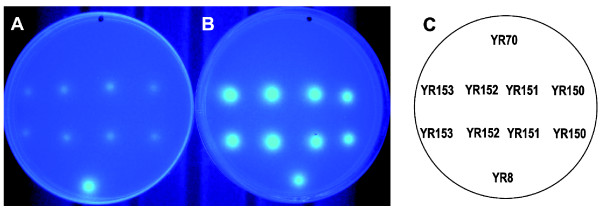
***in vivo *detection of recombinant α-L-rhamnosidase activity**. *S. cerevisiae *cells were grown at 30°C for about 5 h on SD plates containing the artificial substrate MUR. Differences in α-L-rhamnosidase activity between non permeabilized (**A**) and permeabilized (**B**) cells are shown. The names of the strains appear in panel **C**. Representative transformants (YR150-YR153) expressing the *A. nidulans rhaE *gene were tested. Strains harbouring an empty vector (YR70) or a plasmid expressing the *A. aculeatus rhaA *gene (YR8; [27]) were included as controls.

To further confirm that the product of *rhaE *has α-L-rhamnosidase activity, its cDNA was subcloned in a high copy number yeast vector (YEplac195) between the *TDH3 *promoter and the *PGK1 *terminator. The construct was used to transform the *S. cerevisiae *wine yeast strain T_73_-4. Uracil prototrophic transformants (T_73_-4, YEp195_RhaE) were obtained and grown on selective plates containing the artificial substrate 4-methylumbelliferyl α-L-rhamnopyranoside (MUR). Expression of *rhaE *in *S. cerevisiae *resulted in hydrolysis of MUR (Figure 3), confirming that it effectively encodes an α-L-rhamnosidase. As expected, no activity against MUR was found in a transformant (YR70) obtained with the original shuttle vector (T_73_-4, YEp195). Transformants YR150-YR153 containing the expression cassette for α-L-rhamnosidase were thus isolated. Evidence that in *S. cerevisiae *RhaE was not efficiently secreted to the media includes the observation that most of the activity in intact cells was observed after permeabilization of the cell walls with chloroform vapour (Figure 3B).

As RhaE could be secreted in *A. nidulans *via a non-classical mechanism whereas most of the activity in *S. cerevisiae *seems to be entrapped, we examined the cellular location of the recombinant enzyme in *S. cerevisiae*. In general, fungal glycosyl hydrolases bearing a secretion signal peptide in their aa sequences, including the α-L-rhamnosidase RhaA of *A. aculeatus*, are efficiently secreted when expressed in *S. cerevisiae *[27]. Transformant YR150 was cultured for 72 h and aliquots were taken every 24 h. Table 2 shows that at most 17% of the total activity was extracellular or cell-wall bound at any stage of the cultivation whereas intracellular activity was high throughout. In a similar way, the location of RhaE was predominantly intracellular in transformant YR151 (88.1 ± 1.4% intracellular, 8.0 ± 1.3% cell wall bound and 3.8% ± 0.6 extracellular at 72 h). These data suggest that *cis *determinants leading to secretion of RhaE in *A. nidulans *are not functional in *S. cerevisiae*.

**Table 2:** Location of RhaE rhamnosidase activity in recombinant *S. cerevisiae*

Time (h)	OD_600_	Total activity (U ml^-1^)	% Intracellular	% Cell wall	% Extracellular
24	15.1 ± 0.4	0.6 ± 0.02	90.9 ± 0.5	6.0 ± 0.3	3.1 ± 0.2
48	22.9 ± 0.1	1.2 ± 0.1	82.9 ± 1.9	11.2 ± 1.2	5.9 ± 0.7
72	23.0 ± 0.4	1.4 ± 0.3	82.9 ± 2.3	10.7 ± 1.5	6.4 ± 0.9

### Rhamnose induction of *A. nidulans *α-L-rhamnosidase gene expression is subject to carbon catabolite repression independent of CreA

**Figure 4:**
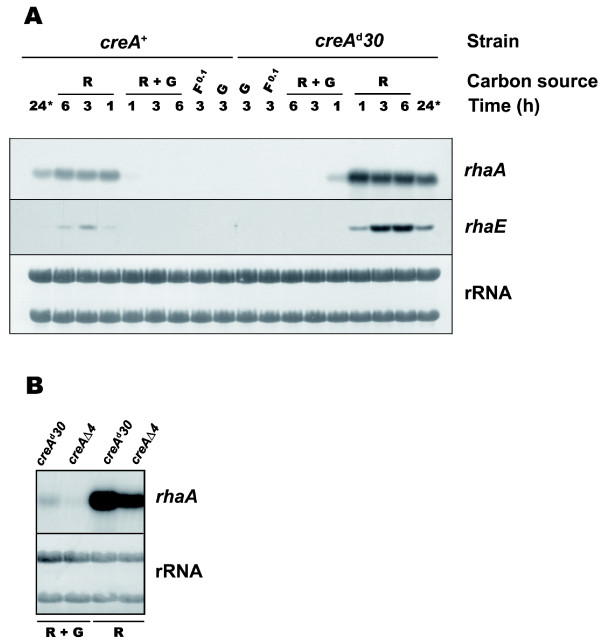
**Glucose repression of *A. nidulans *rhamnosidase genes occurs via a CreA-independent mechanism**. (**A**) Expression levels of *rhaA *and *rhaE *in wild-type (*creA*^+^) and *creA^d^30 *strains during cultivation on 1% w/v rhamnose (R), 0.1% w/v D-fructose (F^0.1^), 1% w/v glucose (G) and 1% w/v rhamnose + 1% w/v glucose (R + G) for the periods indicated. The asterisk (*) indicates growth in 1% w/v rhamnose without transfer. (**B**) Expression levels of *rhaA *in *creA^d^30 *and *creA*Δ4 strains during cultivation on 1% w/v rhamnose (R) and 1% w/v rhamnose + 1% w/v glucose (R + G) for 1 h after transfer. rRNA is shown as a loading control.

We have previously reported that α-L-rhamnosidase activity in *A. nidulans *is induced by rhamnose and repressed by glucose, and that the glucose repression was not relieved by the *creA^d^30 *mutation [8]. Thus we sought to assess whether the results obtained at the enzyme activity level correlated with those of *rhaE *mRNA levels. In addition, we also assessed whether the other *A. nidulans *α-L-rhamnosidase gene *rhaA *[7] co-regulated with *rhaE*. It should be mentioned that the artificial substrate *p*NPR was employed to evaluate α-L-rhamnosidase production in *A. nidulans*, therefore the activity of RhaA was not detected under our original experimental conditions [8,9]. To investigate the role of CreA in the expression of *rhaE *and *rhaA*, northern analyses were performed using both *A. nidulans *wild-type (*creA^+^, biA1*) and a strongly derepressed mutant (*creA^d^30, biA1*) [17]. Total RNAs were isolated from D-fructose grown (0.1% w/v) mycelia transferred in parallel to rhamnose (1% w/v) media containing or lacking glucose (1% w/v), as well as to media containing either D-fructose (0.1% w/v) or glucose (1% w/v) alone. RNAs were also obtained from mycelia grown for 24 h in rhamnose (1% w/v) without transfer. Figure 4A shows that *rhaE *and *rhaA *mRNAs accumulated upon transfer of the wild-type strain to 1% w/v rhamnose but not in mycelia transferred to 0.1% w/v D-fructose (the pre-growth condition) suggesting that rhamnose is required for induction. *rhaE *transcript was detected within 1 h after transfer and accumulated up to 3 h and then decreased; *rhaE *transcript was only observed after 24 h growth in rhamnose upon prolonged exposure of the blot (not shown). Likewise, induction of *rhaA *occurs within 1 h but the transcript level remained constant up to 6 h after transfer; less *rhaA *mRNA was detected after 24 h of growth on rhamnose. Induction of the α-L-rhamnosidase genes thus occurs rapidly after transfer, when the external rhamnose concentration is high (~1%), implying that this does not result from carbon source limitation. Rhamnose-induced expression of both genes in the *creA*^+ ^strain was totally repressed in media containing both rhamnose and glucose or glucose alone (Figure 4A). These results demonstrate that both induction and repression of these genes occurs at the transcriptional level and suggest that they are co-regulated, though RhaA appeared less susceptible to modulation after the initial response to rhamnose.

In the derepressed *creA^d^30 *strain (Figure 4A) induction of *rhaE *and *rhaA *likewise occurred within 1 h of transfer but transcript accumulation was considerably greater than in the wild-type. Contrary to the wild-type, the level of *rhaE *remained stable for at least 6 h after transfer. Interestingly, under inducing-repressing conditions (i.e. 1% w/v rhamnose + 1% w/v glucose), expression of *rhaE *was totally repressed, whilst a low transient expression of *rhaA *was observed 1 h after transfer. That glucose repressed the expression of *rhaE *and *rhaA *in both *creA*^+ ^and *creA^d^30 *genetic backgrounds indicate the lack of a role for CreA in glucose repression of these genes. Moreover, the *creA^d^30 *mutation did not override the need for rhamnose for induction since transcripts were not observed in mycelia grown in 0.1% w/v D-fructose. Interestingly, in the *creA *derepressed mutant the transcript levels of *rhaE *and *rhaA *under inducing (1% w/v rhamnose) conditions were considerably greater than those seen in the wild-type indicating that some derepression occurs when functional CreA is absent and hence suggesting a role for CreA in *rhaE *and *rhaA *expression under inducing conditions. The presence of putative CreA binding sites in the *rhaA *and *rhaE *promoters suggesting direct repression by CreA under certain growth conditions.

To confirm the results with the *creA^d^30 *mutant, we also studied the expression of *rhaA *in a *creA *null mutant (strain *creA*Δ4). Figure 4B shows that glucose repression of *rhaA *expression was not relieved by the *creA*Δ4 mutation, strongly indicating the absence of second-site mutations that could have contributed to the repressed phenotype of the *creA^d^30 *strain, and thus leads weight to the hypothesis of glucose repression of the *rha *genes by a CreA-independent mechanism.

In filamentous fungi numerous genes encoding plant cell wall degrading enzymes have been shown to be subjected to CCR regulated by CreA/Cre1 ([14] and references therein) whereas there are few indications for the existence of CreA-independent mechanisms. Comparison of the levels of expression of reporter constructs in both *creA*^+ ^and *creA^d^30 *backgrounds suggested the existence of both CreA-dependent and CreA-independent mechanisms of CCR regulating *A. nidulans *xylanolytic genes [21,28]. The occurrence in *A. nidulans *of a CCR mechanism independent of CreA has also been suggested for the pectinolytic gene *pelA *[29]. The regulation of the *rhaA *and *rhaE *genes shows similarities to that seen for *pelA *in a derepressed strain (*creA204*) in that the transcripts accumulated to higher levels than in the wild-type when grown under inducing conditions (1% w/v polygalacturonic acid) while glucose repression was not relieved by the loss-of-function *creA204 *mutation. It is possible that the same CreA-independent mechanism regulates these different systems.

### Effect of other carbon sources on α-L-rhamnosidase gene induction

**Figure 5:**
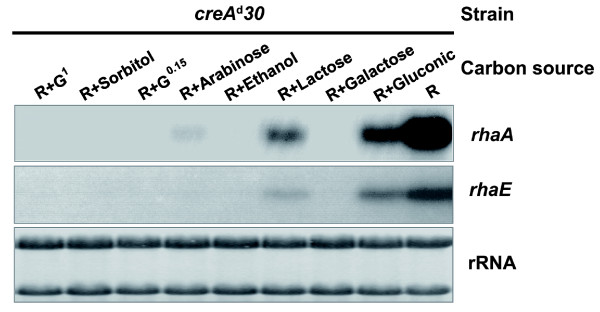
**Repression of rhamnosidase genes in the presence of rhamnose supplemented with different carbon sources**. Northern blot analysis of the expression of the *rhaA *and *rhaE *genes in the *creA^d^30 *strain growing in the presence of rhamnose (R) and different carbon sources. Pregrown mycelia were transferred to inducing medium (1% w/v rhamnose; R) either with or without an equimolar amount of another carbon source and harvested after 3 h of incubation. G^1 ^glucose 1% w/v, G^0.15 ^glucose 0.15% w/v. rRNA is shown as a loading control.

In *A. nidulans*, L-arabinose, ethanol, glycerol and D-lactose are considered to be non-repressing carbon sources whereas glucose and D-xylose are strongly repressing [30]. Concentration is also relevant since at low concentrations the repression by glucose decreases, suggesting that CCR is related to growth rate [31]. We have shown that simultaneous addition of rhamnose with glucose, glycerol, ethanol, L-arabinose or D-lactose resulted in a marked decrease in α-L-rhamnosidase activity [8]. We therefore studied how different carbon sources affect α-L-rhamnosidase gene expression in the *creA^d^30 *mutant. Northern blot analysis was used to study the expression of *rhaE *and *rhaA *in 1% w/v rhamnose-containing media supplemented with equimolar amounts of alternative carbon sources, i.e. D-gluconic acid, D-galactose, D-lactose, ethanol, L-arabinose and D-sorbitol as well as two different concentrations of glucose (0.15 and 1% w/v). The results are shown in Figure 5. The expression profiles of both genes were similar and whilst D-gluconic acid and D-lactose permitted induction of *rhaE *and *rhaA *when mycelia were transferred from 0.1% w/v D-fructose to the inducing media (1% w/v rhamnose) containing these additional carbon sources, they still exerted some degree of repression. A very low level of *rhaA *transcript was also observed in L-arabinose supplemented media. By contrast, D-galactose, ethanol, D-sorbitol and glucose (at high and low concentrations) totally prevented rhamnose induction of both genes. Similarly, only D-lactose and D-gluconic acid allowed rhamnose induction of both genes in the wild-type strain (data not shown). Thus, the sugar classification for CreA-mediated repression does not hold for the CreA-independent repression of α-L-rhamnosidase genes. L-Arabinose, ethanol, D-sorbitol and 0.15% w/v glucose, previously considered less repressive or even 'neutral' carbon sources, all greatly repress *rhaA *and *rhaE *transcription.

Our previous results studying α-L-rhamnosidase production [8] coincide with the current data from northern analyses (Figures 4 and 5) and clearly show that CreA is not solely responsible for CCR of *rhaE *and *rhaA *gene expression, implying that α-L-rhamnosidase genes could be regulated via another repressor(s). It is possible for such a regulator to act directly on α-L-rhamnosidase gene promoters and/or indirectly by repression of the gene(s) encoding rhamnose permeases, rhamnose catabolic enzymes and/or rhamnose pathway specific activator(s). In *S. cerevisiae *it has been shown that glucose repression is mediated in part by the CreA related protein Mig1 and by two homologs (Mig2 and Mig3) which share almost identical zinc fingers and bind similar sites with different affinities [32,33]. In *A. nidulans *and other filamentous fungi only one carbon catabolite repressor CreA has been identified. BLASTP searches using the CreA protein sequence or the sequence of its two zinc fingers failed to find any clear alternative candidate within the zinc-finger family.

### Glucose repression of *rhaA *and *rhaE *correlates with the lack of uptake of rhamnose

**Figure 6:**
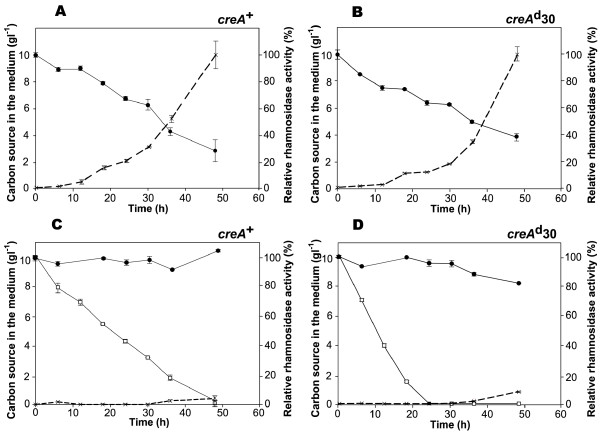
**Inhibition of rhamnose uptake by glucose**. Time-course measurements of rhamnosidase activity and the amount of sugar remaining in the cultures during cultivation of the *A. nidulans *wild-type (*creA^+^*; panels **A **and **C**) and the carbon catabolite derepressed *creA^d^30 *(panels **B **and **D**) strains in MM containing either 1% w/v rhamnose (panels **A **and **B**) or a mixture of 1% w/v rhamnose and 1% w/v glucose (panels **C **and **D**). Rhamnosidase activities are presented as percentages of that observed at 48 h under inducing conditions. The concentrations of rhamnose and glucose in the growth media over time are indicative of the ability to take up these monosaccharides. Symbols: (•) concentration of rhamnose, (□) concentration of glucose, and (X) rhamnosidase activity. Data points are represented as the mean and standard deviation of three independent experiments.

One possible mechanism by which glucose could prevent the induction of *A. nidulans *α-L-rhamnosidase genes would be by reducing the uptake of the inducing sugar rhamnose. To investigate this possibility *A. nidulans *wild-type (*creA*^+^) and mutant (*creA^d^30*) strains were grown for 18 h in the presence of 0.1% w/v D-fructose and mycelia were subsequently transferred to media containing either rhamnose (1% w/v) alone or a mixture of rhamnose (1% w/v) and glucose (1% w/v). Time-course measurements were made of the amounts of the carbon sources remaining in the culture filtrates (determined by high-performance liquid chromatography) and extracellular α-L-rhamnosidase activity (Figure 6). In the same way that the expression profiles of *rhaA *and *rhaE *were similar in the *creA*^+ ^and *creA^d^30 *strains (Figures 4 and 5), the sugar utilization patterns of both strains were quite similar too. When grown in rhamnose as sole carbon source, consumption of this sugar was detectable after 8 h and its concentration decreased more or less linearly with time in both *creA*^+ ^and *creA^d^30 *strains (Figure 6A and 6B respectively). After 48 h of growth about 30% (*creA*^+^) or 40% (*creA^d^30*) of the initial rhamnose was still available, demonstrating the paucity of this sugar as a carbon source compared to glucose which was consumed at a much greater rate and was exhausted after 48 h (Figure 6C and 6D). α-L-Rhamnosidase activity increased in parallel with rhamnose consumption, its initial detection being around 12 h after transfer.

In the mixed sugar cultures glucose was consumed preferentially compared to rhamnose in the wild-type strain and no α-L-rhamnosidase activity was detected (Figure 6C). Hence, a key event in the repression of α-L-rhamnosidase synthesis appears to be the lack of uptake of rhamnose. In concordance with the northern results (see above), CreA does not play a major role in this mechanism of repression since the uptake of rhamnose in the *creA^d^30 *strain was blocked by the presence of glucose, rhamnose only being taken up after the exhaustion of glucose, only after which α-L-rhamnosidase activity could be detected (Figure 6D).

Taken together, these results provide strong indications for the existence of a CreA-independent mechanism of 'inducer exclusion' by which the expression of α-L-rhamnosidase genes, and possibly other genes induced by rhamnose, is regulated. Whether rhamnose permeases are repressed by glucose at the transcriptional level by a factor other than CreA, or controlled post-transcriptionally (e.g. catabolite inactivation of the proteins effecting rhamnose uptake) or whether the rhamnose transport system(s) are competed by glucose - and possibly other carbon sources - is not known at present.

By way of antecedents, glucose repression of the *A. nidulans *proline catabolic cluster genes *prnD *and *prnC *is known to result from inducer exclusion since only the transcription of the proline permease gene (*prnB*) is directly repressed by CreA [34]. CreA-independent mechanisms of inducer exclusion have also been documented, an example of which is the phenylacetic acid (PhAc) uptake system: in the presence of glucose and PhAc, the PhAc transport system is absent in the wild-type as well as in the *creA^d^30 *mutant [35]. Finally, inducer exclusion arising from competitive inhibition of D-xylose uptake by the structurally related sugar glucose has also been suggested to explain at least part of the glucose repression of the xylanolytic gene *xlnA *[28,36]. With regard to the later however, two pieces of data cast doubt on competitive inhibition of inducer uptake as the means of effecting inducer exclusion of rhamnose. Firstly, α-L-rhamnosidase expression was completely repressed by both D-sorbitol and ethanol (Figure 5), carbon sources that are structurally distinct and unrelated to rhamnose. Secondly, the transient *rhaA *response to a mixture of glucose and rhamnose (Figure 4A) suggests that some rhamnose is taken up by the *creA^d^30 *mycelia immediately after medium transfer despite the presence of equimolar amounts of glucose; in the *alc *system the response to induction has been shown to be much faster than the establishment of CCR [36].

**Figure 7:**
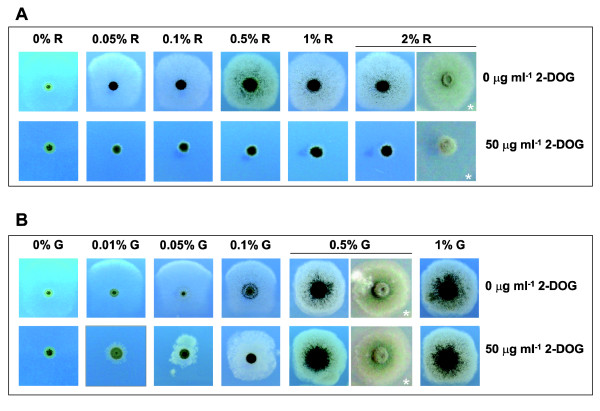
**Rhamnose does not protect from 2-DOG toxicity**. Conidia-derived colonies 3 days after inoculation of the *A. nidulans *reference strain (*biA1*) on MM plates containing different amounts of rhamnose (R) (panel **A**) or glucose (G) (panel **B**) and supplemented (or not) with 50 μg ml^-1 ^of 2-DOG. Photos - except those indicated with an asterisk - were taken against daylight to appreciate better colonies with low hyphal densities.

In *A. nidulans*, it is known that glucose and its non-metabolizable analogue 2-deoxy-D-glucose (2-DOG) are actively taken up by the same transporter(s) [37,38]. Therefore, one easy way to get an idea of whether rhamnose and glucose can compete at the uptake level is to see whether rhamnose can protect against the toxicity of the 2-DOG. Figure 7A shows that increasing amounts of rhamnose (from 0.05 to 2% w/v, equivalent to 2.75 mM to 110 mM respectively) did not reverse the toxicity of 50 μg ml^-1 ^of 2-DOG (0.3 mM). As expected, glucose protection of 2-DOG toxicity was clearly observed under the same experimental conditions (Figure 7B). This result further supports the possibility of glucose effecting rhamnose uptake by repression and/or catabolite inactivation of a transporter(s) instead by competitive inhibition. Experiments are now in progress to characterize the molecular mechanism(s) by which glucose inhibits rhamnose induction in *A. nidulans*.

### Repression by ethanol does not result from the lack of rhamnose transport

**Figure 8:**
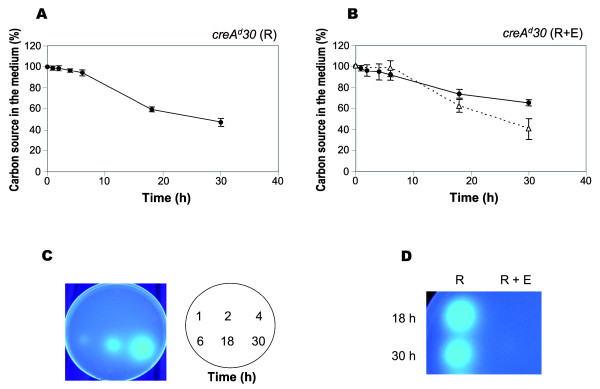
**Co-utilization of rhamnose and ethanol**. Time-course measurement of the amount of rhamnose or ethanol remaining in the cultures during cultivation of the *A. nidulans creA^d^30 *strain in MM containing either 1% w/v rhamnose (R) (Panel **A**) or a mixture of 1% w/v rhamnose and an equimolar amount of ethanol (E) (Panel **B**). Symbols: (•) concentration of rhamnose, (Δ) concentration of ethanol. Data points are represented as the mean and standard deviation of three independent experiments. α-L-rhamnosidase activity was tested by spotting 10 μl of the same cultures on agar plates containing MUR. (Panel **C**) α-L-rhamnosidase activity after transfer to the rhamnose media at the times indicated. (Panel **D**) A sector of a Petri dish showing the lack of α-L-rhamnosidase activity in the mixture cultures.

Bearing in mind the possibility that other carbon sources might negatively affect rhamnosidase gene induction through different mechanisms to that seen for glucose, the effect of ethanol in the uptake of rhamnose was assessed. The *creA^d^30 *strain was grown for 18 h in the presence of 0.1% w/v D-fructose and subsequently transferred to media containing either rhamnose (1% w/v, equivalent to 55 mM) alone or a mixture of rhamnose and ethanol (55 mM each), and the rates of disappearance of rhamnose from each transfer medium along the time were compared. In contrast to the sequential utilization of glucose and rhamnose (Figure 6), simultaneous disappearance of rhamnose and ethanol was observed (Figure 8B), albeit the rate of removal of rhamnose from the media is slightly faster in the absence of ethanol (Figure 8A). Extracellular rhamnosidase activities of these cultures were tested on MUR plates. Accordingly to the northern analyses (Figure 5), in the rhamnose-ethanol media rhamnosidase activity was undetected (Figure 8D) despite rhamnose was being taken up. In the absence of ethanol hydrolysis of MUR became detectable at 6 h (Figure 8C). These results indicate that the mechanism by which ethanol prevents rhamnosidase gene induction and consequently rhamnosidase activity is other than the transport step and thus, CCR of the rhamnosidase system can be achieved by different regulatory mechanisms.

## Conclusions

Using *A. nidulans *as a model system, we show for the first time that the production of α-L-rhamnosidases in eukaryotes is controlled at the transcriptional level by the carbon source (i.e. induction by L-rhamnose, and repression by glucose and other carbon sources). Interestingly, two loss-of-function mutations in the *creA *gene, which encodes the carbon catabolite repression (CCR) factor CreA, resulted in overproduction of *rhaA and rhaE *mRNAs by cells grown in medium containing L-rhamnose as sole carbon source, but had little or no effect on *rhaA and rhaE *mRNA levels in cells grown with glucose plus L-rhamnose as carbon source. We also show that the CreA-independent mechanism of glucose repression correlates with the absence of L-rhamnose uptake. *A. nidulans *α-L-rhamnosidase encoding genes could thus serve as a prototype for fungal genes regulated by a novel inducer exclusion, CreA-independent, mechanism of CCR.

We have also identified the gene (*rhaE*) coding for an α-L-rhamnosidase of oenological relevance that we characterized previously. Phylogenetic analysis shows that this enzyme is the first eukaryotic α-L-rhamnosidase found to be closely related to bacterial rhamnosidases of class A (i.e. *Thermomicrobia *sp. RhmA, *Bacillus *sp. RhaA, *Clostridium stecolarium *RamA and *Lactobacillus acidophilus *RamA) rather than α-L-rhamnosidases from other fungi. The identification of *rhaE *and characterization of its regulation make it possible to design strategies to overproduce the encoded enzyme for industrial applications, as well as identify homologs in other fungi.

## Methods

### Strains and growth conditions

*Escherichia coli *strain DH5α [*endA1, hsdR17, gyrA96, thi-1, relA1, supE44, recA1, ΔlacU169 *(*Φ80 lacZΔM15*)] [39] was used as the recipient strain for cloning experiments and plasmid amplification. The *S. cerevisiae *wine strain T_73_-4 (*ura3*::470*/ura3*::470) [40] was used for functional expression of plasmids bearing the *TDH3*_p_*::rha::PGK1*_t _expression cassettes. The *A. nidulans *wild-type (*biA1; *Glasgow GO51), *creA^d^30 *[17] and Δ*creA *(*creA*-Δ4, *pantoB100*. N.B. offspring of an outcross of the original mutant described by Shroff et al. [41]; M. Flipphi, unpublished data) strains were used.

*E. coli *was grown in LB medium (1% w/v tryptone, 0.5% w/v yeast extract, 1% w/v NaCl) with or without 100 μg ml^-1 ^ampicillin. *S. cerevisiae *strains were grown in YPD-rich medium (1% w/v yeast extract, 2% w/v bacteriological peptone, 2% w/v glucose) or SD-minimal medium (0.17% w/v yeast nitrogen base without amino acids [Difco Laboratories, Detroit, USA], 2% w/v glucose, 0.5% w/v ammonium sulphate, with or without 20 mg l^-1 ^uracil). All media used for *A. nidulans *strains were based on appropriately supplemented medium [40,42] containing 1% w/v of carbon source, unless otherwise specified. For *creA *mutants phosphate was included in the sporulation plates [43]. For solid media 1.5% w/v agar was added.

For transfer experiments mycelial biomass was generated from spores inoculated in minimal medium (MM) supplemented with 5 mM urea as nitrogen source, to which 0.1% w/v D-fructose was added as the sole carbon source. After ~18 h grown at 37°C with orbital shaking at 200 rpm, mycelia were harvested, washed with MM without carbon source and transferred. Induction medium was prepared by substituting D-fructose with rhamnose at 1% w/v, and induction-repression medium by substituting D-fructose with rhamnose plus glucose (1% w/v each). Other carbon sources were added instead of glucose at equimolar amounts.

Sensitivity to 2-DOG was tested by spotting the same number of spores (10^5 ^in 3 μl) on plates containing 5 mM urea as nitrogen source, 50 μg ml^-1 ^of the toxic glucose analogue 2-DOG and different concentrations of glucose or rhamnose. Growth was monitored after incubation for 3 days at 37°C.

Production of α-L-rhamnosidase in *S. cerevisiae *was determined under the following growth conditions: the selected transformants were precultured overnight in SD medium lacking uracil, and cells were then used to inoculate (10^6 ^cells ml^-1^) 50-ml of complete (YPD) medium in 250-ml flasks. Yeast cultures were grown with continuous shaking (200 rpm) at 30°C. Recombinant α-L-rhamnosidase cellular location was determined as described [44].

### DNA manipulations and transformations

*E. coli *plasmid isolation and general DNA manipulations were performed following standard protocols [39]. Transformation of the T_73 _strains was done using lithium acetate to permeabilize the cells as previously described [40,45]. Transformants were selected and maintained on SD plates without uracil. Restriction enzymes, Expand High Fidelity system, and T4 DNA ligase were purchased from Roche Diagnostics and used as recommended by the manufacturer. DNA and cDNA sequencing was carried out using the BigDye Terminator v3.1 Cycle Sequencing Kit (Applied Biosystems, USA) and the ABI PRISM 310 Genetic Analyzer (Applied Biosystems, USA) at the DNA Sequencing Service of the University of Valencia. Chromatograms were analysed using the program Chromas LITE 2.01. Oligonucleotides used in the present study are listed in Additional file 1: Table S2.

### RNA isolation, northern blot analysis and RT-PCR

Total RNA was isolated from powdered mycelia using RNA Plus (Qbiogene, USA) following the manufacturer's instructions. Northern gels were loaded with 15 μg of glyoxylated total RNA per track essentially as described [36,39]. Membranes (Hybond-N, Amersham Biosciences) were stained with 0.03% w/v methylene blue as a loading control. Gene specific fragments corresponding to *rhaE, rhaA *and *gpdA *(350 bp, 385 bp and 610 bp, respectively) were generated by PCR (oligonucleotide pairs: Rha122Ndir_Rha122Nrev; Rha35Ndir_Rha35STOP; gpdAu_gpdAd) and used as templates to generate ^32^P-dCTP radiolabeled probes using the High Prime system (Roche).

Reverse transcription was performed using 1 μg of total RNA, an oligo (dT)_18 _anchor primer and the M-MLV reverse transcriptase (USB). Total RNA was previously treated with RNase-free DNase (Roche) for 30 min at 37°C and the DNase was heat-inactivated afterwards. Amplification of the target cDNA by PCR was performed using internal primers (see below).

### Heterologous expression of the *A. nidulans rhaE *gene

To demonstrate the presence of a full-length α-L-rhamnosidase structural gene, the *S. cerevisiae *wine yeast T_73_-4 was used for its heterologous expression. The open reading frame (ORF) of *rhaE *(GenBank accession no. FR873475) was amplified by PCR from *A. nidulans *cDNA using the primers Rha122dir and Rha122rev and subcloned into pGEM-T-Easy generating pGEM-Rha122. The resulting fragment was ligated between the *S. cerevisiae *glyceraldehyde-3-phosphate dehydrogenase gene promoter (*TDH3*_p_) and the phosphoglycerate kinase terminator (*PGK1*_t_) in the high-copy-number shuttle vector YEplac195 [46] in two subcloning steps. First, a 434 bp *Pst*I*-Sal*I fragment from pGEM-Rha122 was ligated into the *Pst*I*-Sal*I restriction sites of YEp195T that contains the phosphoglycerate kinase terminator and polyadenylation signals (*PGK1*_t_) (isolated from pG-1; [47]); oligonucleotides PGKt-1 and PGKt-2 and the selectable *URA3 *gene, yielding YEp195T-R122. To complete the expression cassette, the *TDH3*_p _*Hin*dIII*-Afl*III fragment (isolated from pR31; [27]) and the rest of the *rha122 *ORF (as a 2154 bp *BspH*I-*Pst*I fragment recovered from pGEM-Rha122) were cloned into *Hin*dIII*-Pst*I restricted YEp195T-R122 to generate YEplac195-Rha122. Fragments obtained by PCR were checked by sequencing to ensure the absence of PCR-induced mutations. Transformants were selected at 30°C on uracil-deficient media. Three of the resultant colonies (YR150-YR153) were randomly picked and selected for further studies.

### α-L-Rhamnosidase assays

α-L-Rhamnosidase activity was measured using an assay based on the hydrolysis of *p*-nitrophenyl α-L-rhamnopyranoside (*p*NPR). Release of *p*-nitrophenol was measured spectrophotometrically at 400 nm. The assay was performed for 15 min at 50°C in a final volume of 250 μl using 1.4 mM substrate in 250 mM McIlvaine buffer (citrate-phosphate buffer) pH 4.0, essentially as described [9]. One unit (U) of enzyme activity was defined as the amount of enzyme that releases 1 μmol of *p*-nitrophenol (from *p*NPR) per minute in the same conditions. Protein concentrations were determined by the Bradford assay [48] using BSA as standard. SD or agar plates supplemented with 40 μM MUR and 1 mM McIlvaine buffer pH 4.0 were used for plate assays of α-L-rhamnosidase activities. After incubation, hydrolysis of MUR was assessed under uv light. Chloroform permeabilization of yeast cells was carried out following the method of Herman and Halvorson [49].

### Protein production, purification and sequence

Protein purification was carried out following a similar protocol to that previously described [9]. Polypeptides remaining in the α-L-rhamnosidase active pool after the final purification step were resolved by SDS-PAGE, manually excised and stored at 4°C in ultrapure water. Protein sequence analyses were performed at the Centro Nacional de Investigaciones Cardiovasculares (CNIC), CSIC, Madrid, Spain. Polypeptides were digested automatically using a Proteineer DP protein digestion station (Bruker-Daltonics, Bremen, Germany) following the protocol described by Schevchenko et al. [50] with minor variations. For peptide mass fingerprinting and LIFT-TOF/TOF spectra acquisition [51] an aliquot of α-cyano-4 hydroxycinnamic acid in 33% v/v aqueous acetonitrile and 0.1% v/v trifluoroacetic acid was mixed with an aliquot of the above digestion solution and the mixture was deposited onto an AnchorChip MALDI probe (Bruker-Daltonics). MALDI-mass spectra were generated using an Ultraflex MALDI TOF/TOF system (Bruker-Daltonics). MALDI-MS and MS/MS data were combined using the BioTools program (Bruker-Daltonics) to search the NCBInr (nonredundant) and SwissProt databases using Mascot software (Matrix Science).

### Sugar analyses

Sugars present in the *A. nidulans *culture filtrates were quantified by high-performance anion-exchange chromatography (HPAEC) using a Dionex (DX500 or ICS 3000) system (Sunnyvale, CA, USA) equipped with a CarboPac PA-1 column and a pulse electrochemical detection unit in the pulsed amperometric detection mode. Separation was performed by isocratic elution with 16 mM NaOH at a flow rate of 1 ml min^-1 ^and the data were analyzed using the Peaknet or the Chromeleon software packages (Dionex). Calibration curves were made using standard solutions of sugars of final concentrations of 0, 1, 2, 5, 10 and 25 mg l^-1^. Ethanol concentrations were measured by an enzymatic method using a kit (Roche).

### Bioinformatic analyses

Protein sequences were obtained using BLAST programs [25] at NCBI (http://www.ncbi.nlm.nih.gov/sutils/genom_table.cgi). Retrieved sequences were subsequently used in additional searches against the *A. nidulans *database at the Broad Institute of MIT and Harvard (http://www.broad.mit.edu/annotation/fgi/). Other bioinformatic tools and software packages were provided by the ExPASy Proteomics Server (http://www.expasy.org/): DNA alignments were done using ClustalW [52]; protein localization was predicted using SignalP 4.0 (http://www.cbs.dtu.dk/services/SignalP/; [53]) or SecretomeP 2.0 (http://www.cbs.dtu.dk/services/SecretomeP/; [22]) using eukaryote or mammalian networks respectively.

### Nucleotide sequence accession number

The *A. nidulans *AN7151 (*rhaE*) cDNA/mRNA sequence has been deposited in the EMBL database under the accession number FR873475.

## Abbreviations

aa: amino acid; CCR: Carbon catabolite repression; 2-DOG: 2-deoxy-D-glucose; GH: Glycosyl hydrolase; MALDI-TOF: Matrix-assisted laser desorption/ionization time-of-flight; MM: Minimal medium; MUR: 4-methylumbelliferyl α-L-rhamnopyranoside; *p*NPR: *p*-nitrophenyl-α-L-rhamnopyranoside; ORF: Open reading frame; RT-PCR: Reverse transcription-polymerase chain reaction; SDS-PAGE: Sodium dodecyl sulfate-polyacrylamide gel electrophoresis.

## Competing interests

The authors declare that they have no competing interests.

## Authors' contributions

JATR, MF and EP carried out the experimental work and were involved in data analyses and interpretation. PM participated in RhaE purification and sugar analyses. MO conceived and designed the study, coordinated the work, was involved in data analyses and interpretation of results and wrote the manuscript. All authors have read and approve the final manuscript.

## Supplementary Material

Additional file 1 **Table S1**. α-L-Rhamnosidases (GH78) for which encoding genes have been experimentally characterized [5-7,54-59]. **Table S2**. Primers used in the present study. Click here for file

Additional file 2 **Figure S1. Nucleotide and amino acid sequences of the *A. nidulans *α-L-rhamnosidase AN7151/*rhaE***. The deduced amino acid sequence of the AN7151/*rhaE *gene product is indicated in green boldface. Introns are shown in red. Sequences used for primer design to amplify cDNA sequences are underlined. An asterisk denotes the stop codon. Underlined amino acids correspond to peptides identified by MALDI-TOF mass fingerprinting. Conserved putative catalytic residues Asp/E458, Glu/D464, Asp/E471 and Glu/E737-experimentally characterized in *Bacillus *sp. RhaB [23]-are highlighted in yellow. The cDNA sequence is deposited in Genbank under the accession number FR873475. Click here for file

Additional file 3 **Figure S2. Amino acid alignment**. Amino acid sequences of *Bacillus *sp. GL1 RhaB (accession no. Q93RE7) and *A. nidulans *AN7151/RhaE rhamnosidases were aligned using ClustalW and default conditions.* indicates identity, : indicates high similarity, and . indicates low similarity. Conserved catalytic residues Asp, Glu, Asp, and Glu-experimentally characterized in RhaB [23]-are highlighted in yellow boldface. Click here for file
