# Supplementary material for: L-Rhamnose induction of Aspergillus nidulans α-L-rhamnosidase genes is glucose repressed via a CreA-independent mechanism acting at the level of inducer uptake
Source: Microb Cell Fact. 2012 Feb 21;11:26. doi: 10.1186/1475-2859-11-26 (PMC3312857; doi:10.1186/1475-2859-11-26)
Supplement: Additional file 1 — Table S1. α-L-Rhamnosidases (GH78) for which encoding genes have been experimentally characterized [5-7,54-59]. Table S2. Primers used in the present study. [file 1475-2859-11-26-S1.DOC]

**Table S1 α-L-rhamnosidases (GH78) for which encoding genes have been experimentally characterized**

Uniprot acc. no Designation^a^ Species References^b^

Q9S3L0 RamA *Clostridium stercorarium* [54]

Q9C1M9 RhaA *Aspergillus aculeatus* [5]

Q9HFW5 RhaB *Aspergillus aculeatus* [5]

Q93RE8 RhaA *Bacillus* sp. GL1 [55]

Q93RE7 RhaB *Bacillus* sp. GL1 [55]

Q6RCI9 RhmA *Thermomicrobia* sp. PRI-1686 [56]

Q6RCI8 RhmB *Thermomicrobia* sp. PRI-1686 [56]

C8VMJ6 AN10277 *Aspergillus nidulans* [7]

A9ZT55 Rhm78 *Aspergillus kawachii* [6]

Q5FJ31 RamA *Lactobacillus acidophilus* [57]

Q88SF8 Ram1/RhaB1 *Lactobacillus plantarum* [57,58]

Q88SF6 Ram2/RhaB2 *Lactobacillus plantarum* [57,58]

EONEV1 Ram *Pediococcus acidilactici* [59]

EONEK0 Ram2 *Pediococcus acidilactici* [59]

^a^ refers to the name given in the corresponding publication.

^b^ chronological order

**Table S2 Primers used in the present study**

Primer Sequence (5´🡒3´) ^a^ Target genes ^b^

Rha122Ndir CATCGGTTTGAGAGTACGCT An *rhaE*

Rha122Nrev GGATGAGAGAAGTGGTCGTT An *rhaE*

Rha35Ndir ACAGAGGGTACAGGAACGAT An *rhaA*

Rha35STOP CCAATGCATATTAGACTCGAGAGGCCTTATAC An *rhaA*

gpdAu CGGAATCTAACGTCGTGTGATGTAGG An *gpdA*

gpdAd CGGGATCCTTGAGCTCGTTCTCAGAAG An *gpdA*

Rha122dir AGCCCTCATGagtCTGTCAATTTCTGGCGTC An *rhaE*

Rha122rev ATTGAGTCGACTCAACCGAGCGTACTCTC An *rhaE*

PGKt-1 CGCGGATCCGTCGACCTAGATAAG Sc *PGK1*

PGKt-2 CCGGAATTCTTGGCCCTCTCCTTTTC Sc *PGK1*

^a^ The sequences underlined sequences indicate restriction sites used for cloning purposes. The ATG, agt (TCG🡒agt silent mutation for Ser) and TCA (anneals to the stop codon) are highlighted in green, grey and red respectively.

^b^ An (*Aspergillus nidulans*); Sc (*Saccharomyces cerevisiae*).
