# Supplementary material for: L-Rhamnose induction of Aspergillus nidulans α-L-rhamnosidase genes is glucose repressed via a CreA-independent mechanism acting at the level of inducer uptake
Source: Microb Cell Fact. 2012 Feb 21;11:26. doi: 10.1186/1475-2859-11-26 (PMC3312857; doi:10.1186/1475-2859-11-26)
Supplement: Additional file 2 — Figure S1. Nucleotide and amino acid sequences of the A. nidulans α-L-rhamnosidase AN7151/rhaE. The deduced amino acid sequence of the AN7151/rhaE gene product is indicated in green boldface. Introns are shown in red. Sequences used for primer design to amplify cDNA sequences are underlined. An asterisk denotes the stop codon. Underlined amino acids correspond to peptides identified by MALDI-TOF mass fingerprinting. Conserved putative catalytic residues Asp/E458, Glu/D464, Asp/E471 and Glu/E737-experimentally characterized in Bacillus sp. RhaB [23]-are highlighted in yellow. The cDNA sequence is deposited in Genbank under the accession number FR873475. [file 1475-2859-11-26-S2.DOC]

atgtcgctgtcaatttctggcgtcacttttgagcaccaccgctctgccttggggattggagagccctctccgcgta 76

**M S L S I S G V T F E H H R S A L G I G E P S P R I 26**

tctcctggcggtttgacggcaccgtctcgaactggacgcaatctgcgtacgagatcgagatcaaccgcgctggaca 152

**S W R F D G T V S N W T Q S A Y E I E I N R A G Q 51**

agcgaacaccttccgcgtcaattcatccgactctgttctcgtgccctggccgagcgacccgctgcaatctggcgag 228

**A N T F R V N S S D S V L V P W**  **P S D P L Q S G E 76**

gaggccactgtccgtgtgcgttcattcggacgcgctaaccagcccgatgcgccgtggtcggaccctgttactgttg 304

**E A T V R V R S F G R A N Q P D A P W S D P V T V E 102**

agccggggctgctggacgaagacgattggcagagcgctgtagcgattgtctctgaccgggagacagaggtcaatgc 380

**P G L L D E D D W Q S A V A I V S D R E T E V N A 127**

tacccatcgaccgatctacttccgcaaggacttcgatgtagatgaggagatcctctccgccagactctatataacg 456

**T H R P I Y F R K D F D V D E E I L S A R L Y I T 152**

gccttgggtgtctacgaggctgagatcaatggccagcccgttggcgaccatgtcctggcgcctggatggcaagcat 532

**A L G V Y E A E I N G Q P V G D H V L A P G W Q A Y 178**

acagccatcgccacgaatacaatacctacgacgtcacggatctgctgcaaaccggcgacaacacgatcggagtcac 608

**S H R H E Y N T Y D V T D L L Q T G D N T I G V T 203**

cgtgggcgagggctggtacgccggcgcgctgacctggtcgatgacgaggaatatctacggggacactctcggtctc 684

**V G E G W Y A G A L T W S M T R N I Y G D T L G L 228**

ctctcactactctctatagccaccgctgatggcaaaacgatctacgtgcccagcgatgagacctggcagtcttcta 760

**L S L L S I A T A D G K T I Y V P S D E T W Q S S T 254**

caggcccgataattgcgtccgagatctataacggcgagacgtacgactccacacaggcaatcgaggggtggtctca 836

**G P I I A S E I Y N G E T Y D S T Q A I E G W S Q 279**

gccgggattcgatgcatctggctggctgggaactcacgaggttaccttcgacaagagcgtcctcgctgcgccagat 912

**P G F D A S G W L G T H E V T F D K S V L A A P D 304**

gcacccgccgtgcgccgggtggaagagcggcggctggagagtgtcttcaagagcgcatctggcaagacggtcctcg 988

**A P A V R R V E E R R L E S V F K S A S G K T V L D 330**

actttggccagaacctcgtcggctggctacgtgtgcgtgtcaaggggccgagaggaagcacaatcagctttgttca 1064

**F G Q N L V G W L R V R V K G P R G S T I S F V H 355**

tactgaaggtctggtatcccgtcgttcttctcttgcggtatggatgtgtagatgctaacgtgaacagtgatggaaa 1140

**T E V M E N 361**

acggcgaagtcgcaacgcgccctctccgcaacgccaaagcaaccgacaacctgacgctctctggcgaagagcaaga 1216

**G E V A T R P L R N A K A T D N L T L S G E E Q E 386**

atgggagccctccttcaccttccacggcttccgctacgtgcaggtgaccggctggcccgaggaaaccgagctcaac 1292

**W E P S F T F H** **G F R Y V Q V T G W P E E T E L N 411**

gccgacagcgtcacggccatcgtgatcaacagcgacatggaacagaccgggttcttcagctgctcgaaccctcttc 1368

**A D S V T A I V I N S D M E Q T G F F S C S N P L L 437**

tgaacaagctccacgagaacatcatctggtcgatgcgtggaaacttcctctccattccgacagattgtccccagcg 1444

**N K L H E N I I W S M R G N F L S I P T D C P Q R 462**

agacgagcgactcggctggacgggcgatatccatgcttttgcgcggaccgcaaacttcatctacgatacttcaggc 1520

**D E R L G W T G D I H A F A R T A N F I Y D T S G 487**

ttcttgcgtgggtggctgagggatgcatactctgagcagctggagaataattgtacggctttccttcttcccaatc 1596

**F L R G W L R D A Y S E Q L E N N Y 505**

cagtcatttcgatgctaaccagagcagacgcacccccttatgttatacccaacgtgctcggccccggctcaccgac 1672

**A P P Y V I P N V L G P G S P T 521**

ctctatctggggcgacgccatcgtcagcgtcccatgggacctcttccaaacctacggcgacaaggccatgctgtcc 1748

**S I W G D A I V S V P W D L F Q T Y G D K A M L S 546**

gagcaatacgctggcgccacagcctggctagataaaggcatcctgcgcaacgaagctgggctctggaaccgttcga 1824

**E Q Y A G A T A W L D K G I L R N E A G L W N R S T 572**

ccttccagtacgcagactggctagaccccctcgcaccaccagacgacccgggtgctgccacaacgaacaagtatct 1900

**F Q Y A D W L D P L A P P D D P G A A T T N K Y L 597**

cgtttcggacgcctacttaatccacagcaccgagctggtcgccaacatttcagcgtatctggaccgccccgacgac 1976

**V S D A Y L I H S T E L V A N I S A Y L D R P D D 622**

gcagagaggtatgctgcggatagggcggatctcacgcgcgcctttcagaaagcctggatctcggccaatgggacgg 2052

**A E R Y A A D R A D L T R A F Q K A W I S A N G T V 648**

ttgcaaacgaaacgcaaacagggctcacactgccgctgtacttcaagctcttcgagcgacctgagcattatacgga 2128

**A N E T Q T G L T L P L Y F K L F E R P E H Y T D 673**

tgcggtatcgaggctcgtcgatatcatcaaagagaacgagtacaaagttggcaccggctttgctggaacacacctc 2204

**A V S R L V D I I K E N E Y K V G T G F A G T H L 698**

ctcggccatacattatccgcctacaacgcctcatcaacattctacaacacccttctgcaggaggatgtccctggat 2280

**L G H T L S A Y N A S S T F Y N T L L Q E D V P G W 724**

ggctgtttcaagtcctcatgaacgggaccacgacctgggagcgctgggatagcatgctggccaacgggtccgtgaa 2356

**L F Q V L M N G T T T W E R W D S M L A N G S V N 749**

Tcccggcgagatgacgagcttcaatcactacgctgtcggaagcgtcggcgcttggatgcacgagaacattggcgga 2432

**P G E M T S F N H Y A V G S V G A W M H E N I G G 774**

Ctgagaccgatagagccgggctggagacggttcgcggtggatgtaaaggttggcggggggttgagcagtgcgcagg 2508

**L R P I E P G W R R F A V D V K V G G G L S S A Q E 800**

agaggtttctgagtccgtacggttctgccgagagtagctgggaggtcagagatggcaagttcatgctgggtgtgaa 2584

**R F L S P Y G S A E S S W E V R D G K F M L G V K 825**

ggtgcctccaaatagtgaggctgttgtcagtcttcctggagcccctacccgtggaaagaaggaggttattgtagga 2660

**V P P N S E A V V S L P G A P T R G K K E V I V G 850**

tcagggatgcatcggtttgagagtacgctcggttga 2696

**S G M H R F E S T L G * 861**

**Additional Figure S1 Nucleotide and amino acid sequences of the *A. nidulans* α-L-rhamnosidase AN7151/*rhaE***

The deduced amino acid sequence of the AN7151/*rhaE* gene product is indicated in green boldface. Introns are shown in red. Sequences used for primer design to amplify cDNA are underlined. An asterisk denotes the stop codon. Underlined amino acids correspond to peptides identified by MALDI-TOF mass fingerprinting. Conserved putative catalylic residues Asp/E458, Glu/D464, Asp/E471 and Glu/E737 - experimentally characterized in *Bacillus* sp. RhaB [23] - are highlighted in yellow. The cDNA sequence is deposited in Genbank under the accession number FR873475.
