# Supplementary material for: L-Rhamnose induction of Aspergillus nidulans α-L-rhamnosidase genes is glucose repressed via a CreA-independent mechanism acting at the level of inducer uptake
Source: Microb Cell Fact. 2012 Feb 21;11:26. doi: 10.1186/1475-2859-11-26 (PMC3312857; doi:10.1186/1475-2859-11-26)
Supplement: Additional file 3 — Figure S2. Amino acid alignment. Amino acid sequences of Bacillus sp. GL1 RhaB (accession no. Q93RE7) and A. nidulans AN7151/RhaE rhamnosidases were aligned using ClustalW and default conditions.* indicates identity, : indicates high similarity, and . indicates low similarity. Conserved catalytic residues Asp, Glu, Asp, and Glu-experimentally characterized in RhaB [23]-are highlighted in yellow boldface. [file 1475-2859-11-26-S3.DOC]

*Bacillus*_Q93RE7_RhaB MAGRNWNASWIWGGQEESPRNEWRCFRGSFDAPASVEGPAMLHITADSRYVLFVNGEQVG 60

*A. nidulans*_RhaE -------------------------------MSLSISG-----VTFEHHRSALGIGEPSP 24

. *:.* :* : : : **

*Bacillus*_Q93RE7_RhaB RGPVRSWPKEQFYDSYDIGGQLRPGVRNTIAVLVLHFGVSNFYYLRGRGGLIAEIEADGR 120

*A. nidulans*_RhaE R------------ISWRFDGTVSNWTQSAYEIEINRAGQANTFRVNSSDSVLVPWPSD-P 71

* *: :.* : .:.: : : : * :* : :.. ..::. :*

*Bacillus*_Q93RE7_RhaB TLAATDAAWRTERLGGQRSNSPRMACQQGFGEVIDARELAEDWALPAFDDGGWAQARSIG 180

*A. nidulans*_RhaE LQSGEEATVRVRSFG--RANQP---------------------------DAPWSDPVTVE 102

:. :*: *.. :* *:*.* *. *::. ::

*Bacillus*_Q93RE7_RhaB PAGTAPWTSLVPRDIPFLTEEKLYPASIQSLSRVKAPKYAAALDLRNQMVPESVNHANPV 240

*A. nidulans*_RhaE PG-------LLDEDDWQSAVAIVSDRETEVNATHRPIYFRKDFDVDEEILS--------- 146

*. *: .* : : . : : :. : :*: ::::.

*Bacillus*_Q93RE7_RhaB SYCGYVATILTLETSGVVTLGFPTGVRGSGVWVDGVLQTEWTGVQPERYYSLNLAAGEHL 300

*A. nidulans*_RhaE -------ARLYITALGVYEAEINGQPVGDHVLAPGWQAYSHRHEYNTYDVTDLLQTGDNT 199

: * : : ** : *. * . * . : * :*::

*Bacillus*_Q93RE7_RhaB VLVDITSSDHGGSSHFAIDSEAAFTLRSPAGDNGVPLATIGTFDQSEYIDHRPGRRMQTD 360

*A. nidulans*_RhaE IGVTVGEGWYAGALTWSMTRNIYGDTLG-----LLSLLSIATADGKTIYVPSDETWQSST 254

: * : .. :.*: ::: : . :.* :*.* * . .:

*Bacillus*_Q93RE7_RhaB HPDYRALPEAAPTAAALEAFASWVKP-FEPSLYTEENVFGSNVWRTLAERRAVPRSVLNA 419

*A. nidulans*_RhaE GPIIASEIYNGETYDSTQAIEGWSQPGFDASGWLGT-------HEVTFDKSVLAAPDAPA 307

* : . * : :*: .* :* *:.* : .. :: .:. . *

*Bacillus*_Q93RE7_RhaB ILPVPEPGVLPVFEDGDCELVIDLGAERSGFIGFELEAPAGTIIDAYGVEYMREG-YTQH 478

*A. nidulans*_RhaE VRRVEERRLESVFKSASGKTVLDFGQNLVGWLRVRVKGPRGSTISFVHTEVMENGEVATR 367

: * * : .**:... : *:*:* : *:: ..::.* *: *. .* *.:* : :

*Bacillus*_Q93RE7_RhaB TYGLDNTFRYICREGRQSYVSP--VRRGFRYLFLTVRGNSAPVKLHEIYIRQSTYPVAEQ 536

*A. nidulans*_RhaE PLRNAKATDNLTLSGEEQEWEPSFTFHGFRYVQVTGWPEETELNADSVTAIVINSDMEQT 427

. :: : .*.:. .* . :****: :* :.: :: ..: . : :

*Bacillus*_Q93RE7_RhaB GSFRCSDALLNATWEISRHTTRLCMEDTFV**D**CPS-Y**E**QVFWVG**D**SRNEALVNYYVFGETE 595

*A. nidulans*_RhaE GFFSCSNPLLNKLHENIIWSMRGNFLSIPT**D**CPQRD**E**RLGWTG**D**IHAFARTANFIYDTSG 487

* * **:.*** * : * : . .*******. *****:: *.****** : * . :::. :

*Bacillus*_Q93RE7_RhaB IVERCLNLVPGSADET---PLYLDQVPSAWSSVIPNWTFFWILACREYAAHTGNEAFAAR 652

*A. nidulans*_RhaE FLRGWLRDAYSEQLENNYAPPYVIPNVLGPGSPTSIWGDAIVSVPWDLFQTYGDKAMLSE 547

::. *. . .. *. * *: . .* . * : . : *::*: :.

*Bacillus*_Q93RE7_RhaB IWPAVKHTLTHYLEHIDDSGLLNMAGWNLLDWAPIDQPNEGIVTHQN-------LFLVKA 705

*A. nidulans*_RhaE QYAGATAWLDKGILRN-EAGLWNRSTFQYADWLDPLAPPDDPGAATTNKYLVSDAYLIHS 606

:.... * : : : ::** * : :: ** * :. : . :*:::

*Bacillus*_Q93RE7_RhaB LRDSRALAAAAGATEEADAFAARADLLAETINAVLWDEEKRAYIDCIHADGRRSDVYSMQ 765

*A. nidulans*_RhaE TELVANISAYLDRPDDAERYAADRADLTRAFQKAWISAN--GTVANETQTGLTLPLYFKL 664

. ::* . .::*: :** *:.::: . . : . : * :*

*Bacillus*_Q93RE7_RhaB TQVVAYLCGVAQGEREAVIEGYLSSPPPAFVQIGSPFMSFFYYEALEKAGRQTLMLDDIR 825

*A. nidulans*_RhaE FERPEHYTDAVSRLVDIIKE---NEYKVGTGFAGTHLLGHTLSAYNASSTFYNTLLQEDV 721

: : .... : : * .. . *: ::.. .: . :*::

*Bacillus*_Q93RE7_RhaB RNYGQMLRYDATTCW**E**MYPNFAENRSNPDMLTRSHCHAWSAAPGYFLGSSILGVKRGADG 885

*A. nidulans*_RhaE PGWLFQVLMNGTTTW**E**RWDSMLANGSVNPGEMTSFNHYAVGSVGAWMHENIGGLRPIEPG 781

.: : :.** ****** : .: * * *. * .: * :: ..* *:: *

*Bacillus*_Q93RE7_RhaB WRTVDIAPQPCDLTWAEGVVPLPQGGHIAVSWEFVSAGKLKLRIEAPEDIEVNVTLPEGI 945

*A. nidulans*_RhaE WRRFAVDVKVGGGLSSAQERFLSPYGSAESSWEVR-DGKFMLGVKVPPNSEAVVSLPGAP 840

** . : : . : *. * ***. **: * ::.* : *. *:** .

*Bacillus*_Q93RE7_RhaB EGEVTQVKYMS---------- 956

*A. nidulans*_RhaE TRGKKEVIVGSGMHRFESTLG 861

.:* *

**Additional Figure S2 Amino acid alignment**

Amino acid sequences of *Bacillus* sp. GL1 RhaB (accession no. Q93RE7) and *A. nidulans* AN7151/RhaE rhamnosidases were aligned using ClustalW and default conditions.* indicates identity, : indicates high similarity, and . indicates low similarity. Conserved catalytic residues Asp, Glu, Asp, and Glu - experimentally characterized in RhaB [23] - are highlighted in yellow boldface.
